# Too True to be Bad: When Sets of Studies With Significant and Nonsignificant Findings Are Probably True

**DOI:** 10.1177/1948550617693058

**Published:** 2017-05-05

**Authors:** Daniël Lakens, Alexander J. Etz

**Affiliations:** 1 Human Technology Interaction Group, Eindhoven University of Technology, Eindhoven, the Netherlands; 2 Department of Cognitive Sciences, University of California, Irvine, CA, USA

**Keywords:** likelihoods, publication bias, statistical inferences, power

## Abstract

Psychology journals rarely publish nonsignificant results. At the same time, it is often very unlikely (or “too good to be true”) that a set of studies yields exclusively significant results. Here, we use likelihood ratios to explain when sets of studies that contain a mix of significant and nonsignificant results are likely to be true or “too true to be bad.” As we show, mixed results are not only likely to be observed in lines of research but also, when observed, often provide evidence for the alternative hypothesis, given reasonable levels of statistical power and an adequately controlled low Type 1 error rate. Researchers should feel comfortable submitting such lines of research with an internal meta-analysis for publication. A better understanding of probabilities, accompanied by more realistic expectations of what real sets of studies look like, might be an important step in mitigating publication bias in the scientific literature.

Psychology journals rarely publish nonsignificant results (Fanelli, 2010). At the same time, it is a mathematical reality that it is often incredibly unlikely (or “too good to be true”) that a set of multiple studies yields exclusively significant results (Francis, 2014; Schimmack, 2012). Here, we use a likelihood heuristic to examine when sets of studies that contain a mix of significant and nonsignificant results are likely to be true, or “too true to be bad.” Hopefully, a better understanding of these probabilities will create more realistic expectations of what sets of studies examining true effects look like. We hope these more realistic expectations will convince researchers to submit lines of research containing mixed results for publication and allow editors and reviewers to evaluate the likelihood of observing mixed results when there is a true effect. When mixed results are observed, researchers might be tempted to search for moderators to explain why a single study did not yield a significant effect or decide to simply not report nonsignificant results. A better understanding of the probability to observe mixed results, accompanied by more realistic expectations of which results to expect, might convince researchers to report a meta-analysis over all performed studies and could be an important step in mitigating publication bias in the scientific literature.

## Probability of Significant Results in a Single Study

The probability of observing a significant or nonsignificant result in a study depends on the Type 1 error rate (α), the statistical power of the test (1−β), and the probability that the null hypothesis is true (cf. Ioannidis, 2005; Wacholder, Chanock, Garcia-Closas, El ghormli, & Rothman, 2004). A study might examine a *true effect*, which means the alternative hypothesis (H_1_) is true (e.g., a correlation that differs from zero) or it might examine a *null effect*, which means the null hypothesis (H_0_) is true (e.g., a correlation that is zero). When performing a statistical test on data, the test result might be statistically significant at a specified α level (*p* < α) or not. Thus, there are four possible outcomes of a study, which are referred to as false positives or Type 1 errors (a significant test result when H_0_ is true), false negatives or Type 2 errors (a nonsignificant result when H_1_ is true), true negatives (a nonsignificant result when H_0_ is true), and true positives (a significant test result when H_1_ is true). When H_0_ is true, the probability of observing a false positive depends on the α level or the Type 1 error rate (e.g., 5%). When H_1_ is true, the probability of observing a true positive depends on the statistical power of the performed test (where an often recommended minimum is 80%), which in turn depends on the α level, the true effect size, and the sample size.

With an α level of 5%, and when H_0_ is true, a false positive will occur with a 5% probability (as long as error rates are controlled, e.g., in preregistered studies) and true negative will occur with a 95% probability. When a test has 80% power, and H_1_ is true, a true positive has a probability of 80%, and a false negative has a probability of 20%. In the long run, researchers will perform a specific percentage of studies where H_1_ is true (with H_0_ being true in the remainder of the studies). If we assume that H_0_ and H_1_ are equally likely (both occur 50% of the time), studies have 80% power, and a carefully controlled α level of 5%, the most likely outcome of a study is a true negative (0.5 × 0.95 = 0.475), followed by a true positive (0.5 × 0.8 = 0.4), followed by a false negative (0.5 × 0.2 = 0.1), followed by a false positive (0.5 × 0.05 = 0.025). This example highlights the importance of examining studies that are a priori relatively more likely to be true, if you want to improve your chances of observing a statistically significant result.

In this article, we will use a likelihood heuristic to examine the evidence in sets of studies that yield mixed results. Continuing the earlier example, we see a single significant result will occur 80% of the time if H_1_ is true and 5% of the time if H_0_ is true. In other words, it is 16 times (0.8/0.05) more likely to have observed a significant result when there is a true effect than when there is no true effect. The larger this relative likelihood, the stronger the evidence our data provide for one hypothesis compared to the other.

We can see the relative likelihood is strongly determined by the low α level but less strongly by the power. For example, increasing the power to 95% increases the relative likelihood from 16 to 19, but inflating the α level to 10% reduces the relative likelihood from 16 to 8. As we will see in the section on *p*-hacking below, mixed results in lines of research can provide strong evidence for the H_1_, but only when Type 1 error rates are controlled (e.g., in preregistered studies, close replications, or appropriate use of Bonferroni or other statistical corrections for multiple comparisons). When error rates are inflated, for example due to multiple comparisons, the evidential value in sets of studies sharply decreases, and very little knowledge can be gained from mixed results, as we will discuss below.

## Probability of Significant Results in Multiple Studies

If we perform multiple studies, we can calculate the binomial probability that we will observe a specific number of significant and nonsignificant findings (see also Ioannidis & Trikalinos, 2007). As an example of a binomial probability, consider the probability of observing *k* heads when flipping a coin *n* times. The observed data are generated by a statistical distribution determined by the unknown parameter θ, which ranges from 0 to 1 and is the true probability of getting heads. A typical question in a coin-flipping example would be to determine the probability of observing *k* number of heads in *n* coin flips, assuming a fair coin (θ = .5).

When multiple studies are performed, the probability of observing *k* statistically significant outcomes in *n* studies can be calculated, assuming either H_0_ is true (and thus θ = α) or H_1_ is true (and thus θ = 1 − β). The probability of observing *k* significant results in *n* studies is:

1 $$\frac{n!}{k!\;(n-k)!}\times {\text{θ}}^{k}\times {\left(1-\text{θ}\right)}^{n-k}.$$

The first term indicates the number of possible combinations of results (e.g., when two of the three studies are significant, either the first, the second, or the third study is nonsignificant, which gives three combinations), which is multiplied by the probability of observing significant results in each of the *k* significant studies, which is then multiplied by the probability of observing nonsignificant results in the remaining nonsignificant studies. This is known as the binomial likelihood function.

**Figure 1.**
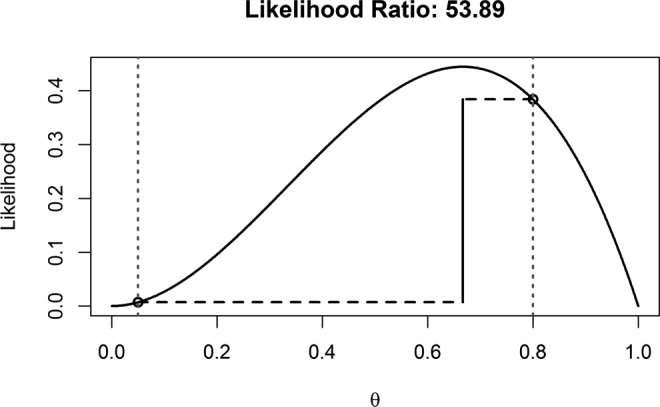
Binomial likelihood curve for two of the three significant results. Vertical lines indicate θ = .05 (the Type 1 error rate assuming H_0_ is true) and θ = .80 (assuming 80% power), with the connecting line visualizing the likelihood ratio.

We can plot the likelihood curve for *k* of *n* significant findings, as a function of θ. In Figure 1, the likelihood curve is plotted for when two significant results are observed in three studies. We can calculate the probability of finding exactly two significant results out of three studies assuming the null hypothesis is true. When H_0_ is true, the probability of significant results equals the α level, and thus when the α level is carefully controlled (e.g., in preregistered studies) θ = .05. When *k* = 2, *n* = 3, and θ = .05, Equation 1 tells us that the probability of finding exactly two significant results in three studies is 0.007 (0.05 × 0.05 × 0.95 = 0.002375, and there are three orders in which two of the three results can be observed, so 0.002375 × 3 = 0.007). To calculate the likelihood assuming H_1_ is true, we need to make an assumption about the power in each study. Let’s provisionally assume all studies were powered at 80% and thus θ = .80. The probability of observing exactly two significant results in three studies, assuming a power of 0.8, is 0.384 (0.8 × 0.8 × 0.2 = 0.128, and with three orders in which two of the three results can be significant, 0.128 × 3 = 0.384).

Both likelihoods at θ = .05 and θ = .80 are highlighted in Figure 1 by the circles on the dotted vertical lines. We can use the likelihood of the data assuming H_0_ or H_1_ is true to calculate the *likelihood ratio*, 0.384/0.007 = 53.89, which tells us the observed outcome of exactly two significant results out of three studies is 53.89 times more likely when H_1_ is true and studies had 80% power, than when H_0_ is true and studies a carefully controlled 5% Type 1 error rate. Likelihood ratios of 8 and 32 have been proposed as benchmarks of moderately strong and strong evidence, respectively (Royall, 1997), which implies that finding two significant results out of the three studies could be considered strong evidence for the H_1_, assuming 80% power. An easy to use *R* script to calculate likelihood ratios and reproduce Figure 1 is available in the Appendix, and a Shiny app to perform these calculations is available at https://lakens.shinyapps.io/likelihood/.

Figure 1 also shows why inflating the Type 1 error rate is problematic for the strength of the evidence in our studies. The higher the actual Type 1 error rate, the lower the likelihood ratio. When you have performed only three statistical tests without correcting for multiple comparisons, the Type 1 error rate has become 1 − (0.95)^3^ = 0.143%. If we recalculate the likelihood ratio, now assuming θ = .143 instead of θ = .05, we see the likelihood ratio has dropped to 7.3, and evidence for the H_1_ is weak. If you do not want to fool yourself that sets of studies with mixed results are strong evidence for H_1_, you need to carefully control error rates.

**Figure 2.**
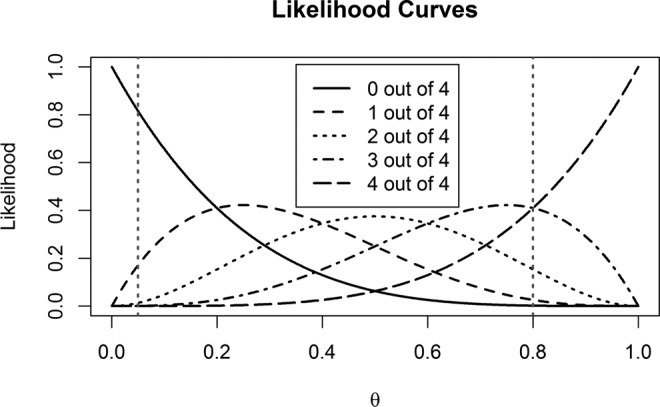
Binomial likelihood curves for the five possible outcomes in four studies, with vertical lines highlighting θ = .05 and θ = .80.

Figure 2 shows the five likelihood curves for the possible outcomes in four studies. The five lines show the likelihood of observing exactly zero, one, two, three, or four significant outcomes. Observing exactly two significant results is most likely when θ = .5, or 50% power, observing 0 significant results is most likely when power is 0%, and finding four significant results is most likely when studies have close to 100% power. When mixed results are observed, the likelihood curves show how each result is relatively more likely when H_1_ is true compared to when H_0_ is true for many reasonable levels of power (e.g., compare the likelihoods at the vertical lines for a 5% Type 1 error rate against the likelihoods assuming 80% power). The maximum likelihood, given the data, is the observed proportion of significant results. In some circumstances, such as when three out of four studies are significant, the likelihood of the data when H_0_ is true is smaller than under almost all possible values of the true power, indicating that almost regardless of your assumptions about the true power of the studies, the outcome is more likely when there is a true effect, than when there is no true effect.

Figure 2 also indicates the long-run frequency with which one will observe a specific set of results. For example, with 80% power, observing four and three significant results out of four studies are 41% likely, two significant studies out of four results is still 15% likely to occur, and one significant result will be found in 3% of the studies (and zero significant results will hardly ever happen). The online Shiny app provides a table with these percentages. In general, any unique outcome of mixed results in *n* studies is more likely than all significant results when power drops below *n*/(*n* + 1)%. For example, when performing four studies, observing only significant results is only the most likely outcome when power is higher than 4/(4 + 1) = 0.8 (or 80%). Note that this is the most likely *unique* outcome, but with a long-run frequency of 41%, it is not *overall* the most likely outcome, since zero, one, two, or three significant results will be observed 59% of the time, and mixed results are overall most likely. Given that many estimates of the average power in psychology suggest the true power of studies is somewhere around 50% (e.g., Fraley & Vazire, 2014), Figure 2 drives home the point that mixed results should have been very likely in the past literature (unless Type 1 errors were severely inflated). But in future studies, mixed results will continue to be very likely. For example, performing six studies with a respectable 89% power in each study is still more likely to yield mixed results than only significant results. The Shiny app allows users to perform these calculations easily.

## Likelihood Ratios

The likelihood ratio is a comparison of how well two hypotheses (in this case, H_0_ and H_1_) predict the data. Let’s first consider the likelihood ratio in a single study with an α level of 5%, 80% power when H_1_ is true, and a 50% prior probability that H_0_ is true. In the long run, the probability of observing a false positive in this scenario is 2.5% (an α of 0.05 × 0.50 probability of H_0_ being true) and the probability of observing a true positive is 40% (power of 0.80 × 0.50 probability of H_1_ being true). After having observed a significant finding, the odds that this result was sampled from H_1_ versus H_0_ change from 1-to-1 (or 50–50) prior odds to posterior odds of 0.40/0.025, or 16-to-1. The long run probability of observing a true negative is 47.5% ([1−0.05] × 0.5) and the probability of observing a false negative is 10% ([1−0.8] × 0.5). After having observed a nonsignificant finding, the odds that the result was observed from H_1_ versus H_0_ change from 1-to-1 prior odds to posterior odds of 0.1/0.475, or 1-to-4.75.

Likelihood ratios can be easily generalized to multiple studies by simple multiplication. For the case above, each observed significant result gives a likelihood ratio of 16-to-1 in favor of H_1_, and every nonsignificant result gives a likelihood ratio of 1-to-4.75 in favor of H0. If two significant results (16:1 × 16:1 = 256:1) and one nonsignificant result (1:4.75) are observed, this gives an overall likelihood ratio of 256/4.75 = 53.89 for H_1_ versus H_0_ (see Figure 1). This multiplicative tally could continue for as many studies the researcher decides to run, and the order in which these results are obtained does not affect the likelihood ratio (Royall, 1997). If researchers assume the power of each individual study varies, likelihoods can be calculated for each individual study and easily combined using simple multiplication.

We can now address one of the main insights we wish to communicate. In sets of studies, the likelihood ratio in favor of H_1_ versus H_0_ after observing a mix of significant and nonsignificant findings can become surprisingly large. Even though the evidence appears to be mixed, there is actually strong evidence in favor of a true effect. For example, when a researcher performs six studies with 80% power and a 5% α level and finds three significant outcomes and three nonsignificant outcomes, the cumulative likelihood ratio is convincingly large at 38-to-1 in favor of H_1_ to consider the set of studies strong evidence for a true effect. With 1-to-1 prior odds, the probability that H_1_ is true given these data are over 97% (38/[38 + 1] = .974). When four or five of the six studies are significant, the likelihood ratio increases by orders of magnitude, and the probability that H_1_ is true increases to more than 99%, even with small prior odds. The presence of some nonsignificant findings is to be expected, and no reason to doubt the strength of the evidence in favor of H_1_ (as long as the Type 1 error rate is carefully controlled). Intuitively, researchers might not feel convinced by a set of studies where three out of six results were statistically significant. But if we do the math, we see that such a set of studies can be very strong evidence in favor of a true effect. A better understanding of these probabilities might be an important step in mitigating the negative effects of publication bias.

## α Levels, Power, *p* Hacking, and Prior Probabilities

The examples so far have assumed an α level of 5%, 80% power, and an equal probability that H_0_ and H_1_ are true (or a noninformative prior). Below, we will discuss how different values for these parameters influence the binomial likelihood curves and likelihood ratios.

### α Level and Power

In general, the lower the α level, the lower the chance of a false positive, and thus studies with lower α levels that contain significant findings will have relatively more favorable likelihood ratios than studies with a higher α level, all else being equal. This can be seen in the likelihood curves in Figure 2 or by reducing the α level in the online Shiny app. For mixed results, lower α levels (e.g., θ = .01) lead to larger likelihood ratios in favor of H_1_. For example, when α is lowered from 0.05 to 0.01, and assuming 80% power, the 16-to-1 likelihood ratio for a significant result when α = 0.05 increases to an 80-to-1 likelihood ratio when α = 0.01, and the 1-to-4.75 ratio after a nonsignificant result slightly increases to 1-to-4.95.

The true effect size is unknown, and therefore the power of each study is unknown, so it is advisable to examine the likelihood across a range of possible levels of power. Ideally, researchers can consider a smallest effect size of interest, which serves as a lower limit for effect sizes (and thus power estimates) that are considered either theoretically or practically relevant. Some researchers have suggested 33% power is a reasonable lower bound against which to compare observed results (e.g., using a *p curve,* Simonsohn, Nelson, & Simmons, 2014), based on the idea that power below 33% would make it very difficult to distinguish signal from noise in hypothesis tests. There will always be uncertainty with respect to the true power of studies, but in larger sets of studies, sets of mixed results will be convincing evidence for H1 for a wide range of power estimates.

### *p* Hacking

A related point of discussion is how the likelihood ratios we discuss here are affected by inflated error rates due to practices that increase the false positive rate. A simple example is optional stopping. If a researcher analyzes the results repeatedly as additional data comes in, and only stops when a result is statistically significant, the Type 1 error rate can drastically increase (Lakens, 2014). A less-recognized consequence of practices that inflate Type 1 error rates when the null hypothesis is true is that they also increase power when H_1_ is true (Salomon, 2015). If we compute the achieved power for a two-sided independent *t* test with 50 participants in each condition, an effect size of 0.5, and an α level of 0.05, we have 70% power. If we allow the α level to be inflated to 0.2 (20%) or 0.5 (50%), power also increases to 89% or 97%. With a controlled α of 0.05 and 70% power, observing one significant effect yields a likelihood ratio of 14-to-1, which is informative, but inflating the Type 1 error rate to 20% (and power to 89%) or 50% (and power to 97%) reduces the likelihood ratio to 3.56-to-1 or 1.94-to-1, which provides only very weak evidence for H_1_. With extreme *p* hacking, even long chains of significant studies become uninformative about the underlying truth of the world. We feel this consequence lines up with the intuitions many researchers have, namely, that in the presence of gratuitous *p* hacking, we cannot learn much from the data.

### Prior Probability of H_0_

The strength of the evidence is independent of the prior odds we assign to the hypotheses, and likelihood ratios have therefore been recommended as precise and objective measures of the strength of evidence (Royall, 1997). Nevertheless, prior probabilities necessarily influence posterior probabilities, and whether one finds the strength of evidence in the data convincing support for H_1_ depends on one’s prior belief that H_0_ is true (Savage, 1962). Although by no means intended to replace a formal Bayesian analysis of the data, posterior odds can easily be calculated based on the likelihood ratio calculated from binomial probabilities.

Let’s assume that instead of H_0_ and H_1_ being equally likely (50–50%), we initially believe H_0_ is 24 times more likely to be true than H_1_. This means that a priori, we are very skeptical about a true effect, and we believe the probability that H_0_ is true is 96%, with only a 4% probability that H_1_ is true. In this scenario, the probability of observing a true positive is our prior belief that H_1_ is true multiplied by the power, 0.04 × 0.8 = 0.032, and the probability of observing a false positive is 0.96 × 0.05 = 0.048. Because H_1_ was a priori extremely unlikely, after one significant result the odds that a significant result was drawn from H_1_ rather than H_0_ are 0.032-to-0.048 or 1-to-1.5—which means we still believe H_0_ to be more likely. Our main point here is to highlight that likelihood ratios provide relative evidence and that likelihood ratios that favor H_1_ over H_0_ do not immediately imply H_1_ is most likely to be true. Since these binomial probabilities ignore information (i.e., they treat all *p* < .05 the same, regardless of whether *p* = .049 or *p* = .00001), a formal Bayesian analysis is the best approach when interested in quantifying one’s posterior belief in a hypothesis (e.g., see Etz & Vandekerckhove, 2016).

## General Discussion

When performing multiple studies that test the same hypothesis, it becomes increasingly likely that not all studies will reveal a statistically significant effect. Using a likelihood approach, we aimed to show that observing mixed results becomes increasingly likely when multiple studies are performed. At the same time, the probability that H_1_ tested in a set of studies is true, even though one or more studies yielded nonsignificant results, can be surprisingly large as long as Type 1 error rates are carefully controlled. Thus, unless studies can reasonably be assumed to have exceedingly high power (e.g., the effect is known to be very large or the sample sizes are very large), we should expect to see mixed results in lines of research. When such mixed results are observed, and error rates were carefully controlled, the data are often much more likely to occur when there is a true effect than when there is no true effect, and such mixed results could be interpreted as support for H_1_.

Nonsignificant findings in lines of research are rarely published (Fanelli, 2010), and with widespread publication bias, it is very difficult to draw quantitative conclusions from the scientific literature. It is our hope that researchers become more inclined to submit nonsignificant findings for publication when they have a better understanding of the evidential value in lines of research with mixed results. Publishing all performed studies in lines of research will reduce publication bias, and increase the informational value of the data in the scientific literature. Expecting all studies in lines of research to be statistically significant is not reasonable (Schimmack, 2012), and it is important that researchers develop more realistic expectations if they are to draw meaningful inferences from lines of research.

The calculations presented here are based on the probability of observing true positives, false positives, true negatives, and false negatives and follow the logic outlined in earlier work by Wacholder, Chanock, Garcia-Closas, El ghormli, and Rothman (2004) and Ioannidis (2005). Where Wacholder et al. focus on identifying when individual studies are unlikely to be studying true effects, and Ioannidis focuses on when bias leads to lines of positive research findings that are probably not true effects, we focus on when a line of research with a mix of significant and nonsignificant studies is likely to be investigating true nonzero effects. Where previous work highlighted the need to be skeptical of the published literature (e.g., Ioannidis & Trikalinos, 2007), we aim to provide researchers, reviewers, and editors with a heuristic to evaluate when there is no need to be overtly skeptical about mixed results.

Researchers have been exposed to a literature that is about as representative of real science as porn movies are representative of real sex. Educating researchers about binomial probabilities and likelihood ratios is a straightforward way to develop more realistic expectations about what research lines that contain evidential value in favor of H_1_ look like.

We do not mean for these binomial probabilities to be used to communicate the probability that hypotheses are true, for which formal Bayesian analyses of the observed data are needed. Nor are these binomial probabilities meant to provide estimates of the effect size or the probability of observing a specific meta-analytic effect size, assuming the H_0_ is true, for which estimation and meta-analysis is the best tool. The likelihood ratios can be used as a heuristic by researchers who want to decide whether a set of observed studies is more probable assuming that H_1_ is true (given a specific assumption about the power in the line of research) than when H_0_ is true. Researchers should feel comfortable to submit lines of studies with a mix of significant and nonsignificant outcomes for publication instead of selectively reporting significant outcomes, and reviewers and editors might feel confident that the resulting inferences, however analyzed, are much less biased than lines of research with exclusively significant results.

We repeat recent recommendations to rely more strongly on meta-analytic evaluations of research lines (Braver, Thoemmes, & Rosenthal, 2014; Fabrigar & Wegner, 2016). It is possible that cumulative evidence across studies provides support for an effect, even when a more dichotomous evaluation of significant versus nonsignificant results suggests that the pattern of results is not very likely. This can happen because this heuristic, like all heuristics, ignores some information. Effectively, this heuristic reduces the information in the studies to whether the data fall in a region where they are significant or not, irrespective of where exactly the data fall. We especially lose information when the set of studies contains low nonsignificant *p* values. For example, Tuk, Zhang, and Sweldens (2015) examined the effects of ego depletion and observed only two statistically significant results in 18 experiments. In the best case (using the observed average power in the studies of 11%), the likelihood ratio provides 1.7-to-1 support in favor of the H_1_ (.11/.05). Similarly, Zhang, Lakens, and IJsselsteijn (2015) performed three studies (including one preregistered replication) and observed only one statistically significant result, which has a maximum likelihood ratio of 3.28-to-1. Nevertheless, in both cases, a meta-analysis incorporating all the information in the cumulative data revealed that the observed data supported H_1_.

Throughout this article, there has been a prominent distinction between significant and nonsignificant findings. The use of such a dichotomy is an intuitive way to explain which outcomes are likely to be observed in lines of research (cf. Ioannidis, 2005; Wacholder et al., 2004), before the data are collected. After the data are collected, looking only at significant versus nonsignificant throws away much of the information contained in the sample, so we encourage authors, editors, and reviewers to use these ratios merely as a heuristic rather than as a formal analysis tool. A proper analysis should take into account all the information from the samples, using either a Bayesian analysis, or a cumulative meta-analytical approach to statistical inferences. What is most important is to prevent researchers from drawing inferences from a biased subset of all data. It is tempting to explain away nonsignificant results in a line of studies by minor differences in the method, even when random variation is a much more likely explanation. By understanding which patterns in sets of studies are to be expected in the presence of true effects, researchers might feel more comfortable in drawing inferences over all performed studies, regardless of their significance level.

## Conclusion

People are notoriously bad at thinking about probabilities in general (Tversky & Kahneman, 1971) and the power of studies in particular (Bakker, Hartgerink, Wicherts, & van der Maas. 2016). A better understanding of probabilities is essential to evaluate lines of research. Researchers have recently been reminded that sets of studies that contain only significant findings can be too good to be true, but they lack a clear explanation of when a set of findings that contain significant and nonsignificant results is too true to be bad. If we want to reduce publication bias, it is important that authors, reviewers, and editors have a more realistic understanding of when lines of studies with mixed results are relatively more likely when the H_1_ is true than when the null hypothesis is true. We must embrace multistudy articles with mixed results if we want our scientific literature to reflect reality.

## Appendix

#Calculate the likelihood ratio----
n<-3 #set total studies
x<-2 #set total significant studies
H0 <- 0.05 #specify H0 (alpha)
H1 <- 0.8 #specify H1 (power)
LR<-round(max(dbinom(x, n, H0)/dbinom(x, n, H1), dbinom(x, n, H1)/
dbinom(x, n, H0)), digits=2)
theta<- seq(0,1, len=1000)
like <- dbinom(x, n, theta)
#png(file=‘‘Fig1LikRatio.png’’, width=3000, height=2000, res = 500)
plot(theta, like, type=‘l’, xlab=expression(theta), ylab=‘Likelihood’, lwd=2)
points(H0, dbinom(x, n, H0), lwd=2)
points(H1, dbinom(x, n, H1), lwd=2)
segments(H0, dbinom(x, n, H0), x/n, dbinom(x, n, H0), lty=2, lwd=2)
segments(H1, dbinom(x, n, H1), x/n, dbinom(x, n, H1), lty=2, lwd=2)
segments(x/n, dbinom(x, n, H0), x/n, dbinom(x, n, H1), lwd=2)
abline(v=0.05, col=‘‘gray40’’, lty=3, lwd=2)
abline(v=0.8, col=‘‘gray40’’, lty=3, lwd=2)
title(paste(‘Likelihood Ratio:’, LR))
#dev.off()
dbinom(x, n, H1)#probability of x out of n sig studies, given H1 power
dbinom(x, n, H0)#probability of x out of n sig studies, given alpha
LR #Likelihood Ratio
